# Case Report: From teratoma to adenocarcinoma: molecular insights into somatic-type malignancy in testicular germ cell tumors - two case reports and review of the literature

**DOI:** 10.3389/pore.2025.1612227

**Published:** 2025-10-28

**Authors:** Tímea Rozsvai, Boglárka Pósfai, László Torday, Emőke Borzási, György Lázár, Judit Oláh, Bence Radics, István Előd Király, István Papos, Márton Balázsfi, Zsombor Melegh, Levente Kuthi, Anikó Maráz

**Affiliations:** ^1^ Department of Oncotherapy, Albert Szent-Györgyi Medical School, University of Szeged, Szeged, Hungary; ^2^ Department of Pathology, Albert Szent-Györgyi Medical School, University of Szeged, Szeged, Hungary; ^3^ County Center of Clinical Oncology and Radiotherapy, Békés County Central Hospital, Gyula, Hungary; ^4^ Department of Surgery, Albert Szent-Györgyi Medical School, University of Szeged, Szeged, Hungary; ^5^ Department of Urology, Albert Szent-Györgyi Medical School, University of Szeged, Szeged, Hungary; ^6^ Department of Neurosurgery, Albert Szent-Györgyi Medical School, University of Szeged, Szeged, Hungary; ^7^ Department of Surgical and Molecular Pathology, Tumor Pathology Center, National Institute of Oncology, Budapest, Hungary; ^8^ Department of Pathology and Experimental Cancer Research, Semmelweis University, Budapest, Hungary; ^9^ HUN-REN-ONKOL-TTK-HCEMM Oncogenomics Research Group, National Institute of Oncology, Budapest, Hungary

**Keywords:** oncology, postpubertal-type teratoma, somatic-type malignancy, NGS, testis

## Abstract

Testicular germ cell tumors (TGCTs), though typically responsive to therapy, may rarely develop somatic-type malignancy (STM), a transformation associated with poor prognosis and chemoresistance. This study presents two cases of postpubertal-type teratoma with intestinal-type adenocarcinoma as STM, offering insights into their clinical, histopathological, immunophenotypic, and molecular profiles. The first patient, a 63-year-old male, presented with pulmonary and retroperitoneal metastases and underwent orchiectomy, revealing an intratesticular intestinal-type adenocarcinoma. Molecular testing confirmed 12p overrepresentation and pathogenic mutations in *CTNNB1*, *STK11*, and *MDM2*. The second patient, initially diagnosed at age 35 with a mixed TGCT, developed STM as a late recurrence 16 years post-orchiectomy, manifesting as a retroperitoneal mass with vertebral invasion. Histology again confirmed intestinal-type adenocarcinoma, and molecular testing revealed amplification of *ERBB2*, *KRAS*, along with mutations in *TP53* and *PIK3CA*. Both cases were managed with capecitabine-oxaliplatin plus bevacizumab, followed by maintenance therapy, achieving disease stabilization for at least 9 months. These cases illustrate the diagnostic and therapeutic complexities of STM, particularly with adenocarcinoma morphology that may mimic primary gastrointestinal neoplasms. Accurate diagnosis required exclusion of alternate primary sites and demonstration of chromosome 12 aberrations using FISH and next-generation sequencing. Our findings emphasize the importance of long-term follow-up in TGCT patients, particularly those with teratomatous elements, and highlight the value of cytogenetic and molecular profiling in confirming STM and identifying potential therapeutic targets. Given the rarity of STM, especially in metastatic or recurrent settings, there is an urgent need for standardized diagnostic protocols and evidence-based treatment strategies. These cases support the use of tumor-specific chemotherapy regimens guided by the histological and molecular characteristics of STM.

## Introduction

Testicular germ cell tumors (TGCTs) are the most common solid neoplasms in young adults and generally have a favorable prognosis, even in metastatic cases [1, 2]. Despite their histological diversity, including seminoma, embryonal carcinoma, yolk sac tumor, choriocarcinoma, and postpubertal-type teratoma, these tumors originate from a common pre-neoplastic lesion and share key genetic traits [3].

A hallmark of TGCTs is their association with germ cell neoplasia *in situ* (GCNIS), characterized by atypical, gonocyte-like cell proliferation within the seminiferous tubules [2, 3]. Additionally, chromosomal abnormalities, particularly isochromosome 12p (i12p), are consistently observed across these tumor subtypes [2, 3].

Approximately 3%–6% of TGCTs exhibit somatic-type malignancy (STM), which can manifest as various forms of sarcoma, carcinoma, hematologic malignancies, or combinations thereof [4]. STM may arise in the primary tumor or metastatic sites and is clinically significant due to its association with a more aggressive disease course and resistance to platinum-based chemotherapy [3].

In this paper, we present two cases of metastatic postpubertal-type teratoma with STM and provide a comprehensive review of pathological features, genetic background, and current treatment strategies.

## Case presentations

Table 1 summarizes the clinical, pathological, immunohistochemical, and genetic characteristics of the patients investigated.

**TABLE 1:** Clinicopathological features of the tumors investigated.

Patient	Age (years)	Histology of STM	Other TGCT	Localization	Size (mm)	Immunohistochemical features	Chr 12 FISH	Molecular genetic data
1	63	Intestinal-type adenocarcinoma	No	Primary testicular	56	SALL4+, CK7-, CK20+, CDX2++++, SATB2-, FOXA2-, Cadherin17++++, Inhibin+, MelanA-, HER2-, pMMR	12p gain	Mutation: *CTNN1B*, c.101G>A, VAF: 28.61% *STK11*, c.924_949delGTTCCGGAAGAAACATCCTCCGGCTG, VAF: 61.37% Amplification: *MDM2*, CN: 98.4 *EGFR*, CN: 7.96 *HNF1A*, CN: 4.74 *CCND2*, CN: 4.48 *CDKN1B*, CN: 4.06
2	52	Intestinal-type adenocarcinoma	No	Metastasis to the retroperitoneum (17 years later)	70	CK20++++, CK7++, panCK++++, TTF1-, SATB2++++, OCT4-, SALL4-, CD30^−^, HER2: 3+, pMMR	12p gain	Mutation: *TP53*, c.514G>T, VAF: 79% *PIK3CA*, c.1635G>T, VAF: 29.6% Amplification: *ERBB2*, CN: 53.6 *KRAS*, CN: 4.22 *CCND2*, CN: 4.53 *CDKN1B*, CN: 5.17

STM indicates somatic-type malignancy; TGCT, testicular germ cell tumor; chr 12, chromosome 12; FISH; fluorescent *in situ* hybridization; pMMR, proficient mismatch repair status; VAF, variant allele frequency; CN, copy number.

### Patient 1

The clinical examination of a 63-year-old male patient began in March 2023 due to enlargement of the left testicle. His medical history was unremarkable, with no known previous illnesses or surgeries, and he was not on any regular medications. Testicular ultrasound was performed, raising suspicion of a malignant lesion in the left testicle. Tumor markers, including LDH (199 U/l), AFP (3.41 ng/mL), beta-HCG (<0.6 IU/L) were within the normal range.

**FIGURE 1:**
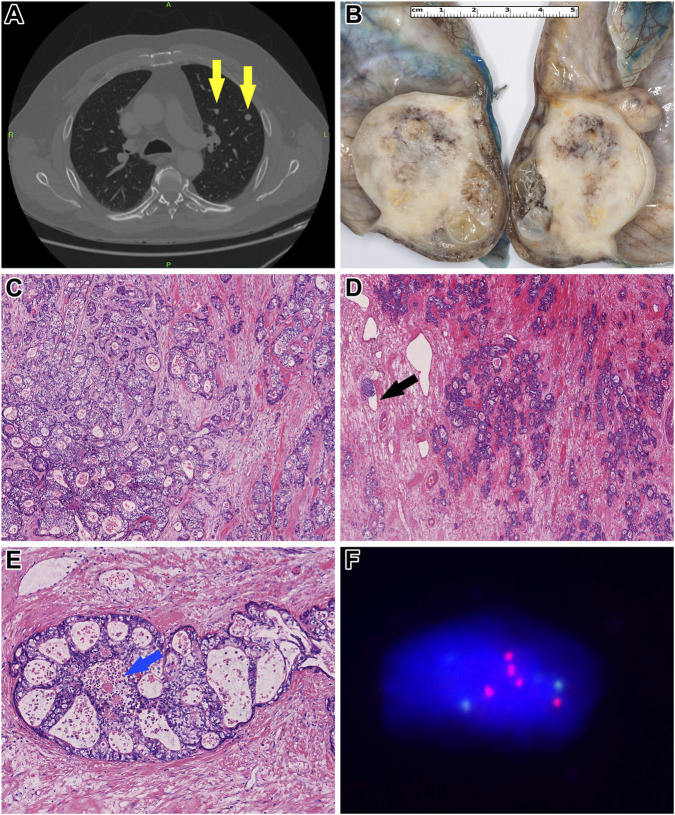
Morphological and molecular characteristics of *Patient 1’s* tumor. **(A)** Contrast-enhanced chest CT revealed multiple metastatic foci (yellow arrows). **(B)** Gross examination of the orchiectomy specimen showed a heterogeneous gray-whitish tumor mass. Notably, the normal architecture of the testis and epididymis was entirely effaced by the tumor. **(C)** Histologically, the tumor cells exhibited pale eosinophilic to optically clear cytoplasm, accompanied by a moderate degree of cytological atypia (HE, x200). **(D)** Within a desmoplastic stroma, the neoplastic cells formed atypical, confluent tubules. Occasional lymphovascular invasion was identified (black arrow) (HE, x100). **(E)** In other regions, a cribriform growth pattern was observed, with central areas showing so-called “dirty” necrosis [blue arrow] (HE, x400). **(F)** Fluorescence *in situ* hybridization analysis demonstrated overrepresentation of chromosome 12. Green signals indicate the centromere of chromosome 12, while red signals correspond to its short arm.

A chest, abdominal, and pelvic CT scan revealed multiple solid nodules measuring 4–14 mm in both lungs, raising the possibility of metastases (Figure 1A). Additionally, a 12 mm pathological lymph node infiltrating the surrounding fat tissue was identified in the left infrarenal region. Subsequently, the patient underwent a left-sided orchiectomy in April 2024. Gross examination of the surgical specimen revealed a 46x36x32 mm cystic mass occupying the testicular parenchyma (Figure 1B). On the cut surface, no intact testicular or paratesticular parenchyma was identified.

Histopathological analysis showed a tumor composed of columnar cells with clear or pale eosinophilic cytoplasm and large nuclei (Figure 1B). The architecture was glandular, resembling an intestinal-type adenocarcinoma typically seen in the colorectum (Figures 1C, D). The tumorous glands contained mucin and exhibited “dirty necrosis” within their lumina (Figure 1E). No other germ cell tumor components, including yolk sac tumor or “classic” postpubertal-type teratoma, were present. Due to complete destruction of the testicular parenchyma, the presence of GCNIS could not be assessed. Immunohistochemically, the tumor cells showed diffuse positivity for CDX2 and Cadherin-17, while CK20 and SALL4 were only focally positive. In contrast, CK7, OCT4, Glypican-3, and FOXA2 were completely negative. The expression of mismatch repair (MMR) proteins, including MLH1, MSH2, MSH6, and PMS2, was retained, and HER2 expression was absent.

To investigate the presence of i12p, fluorescent *in situ* hybridization (FISH) was performed using the ZytoLight SPEC KRAS/CEN 12 Dual Color Probe (ZytoVision GmbH, Bremerhaven, Germany). Additionally, panel-based next-generation sequencing (NGS) was conducted using the Oncomine Comprehensive Assay v3 panel (Thermo Fisher Scientific, Waltham, MA, USA). FISH confirmed 12p overrepresentation (Figure 1F), which was further supported by copy number alterations in *MDM2*, *HNF1A*, *CCND2*, and *CDKN1B* genes detected in the NGS data. Also, the analysis detected pathogenic *CTNNB1* and *STK11* mutations.

Based on histomorphology, immunohistochemistry, and molecular pathology findings, the diagnosis of postpubertal-type teratoma with STM, specifically intestinal-type adenocarcinoma, NOS, was established. Tumor cells had invaded the hilar soft tissue and were found surrounding entrapped normal paratesticular tubules, leading to a local pathological stage classification of pT2. Additionally, extensive lymphovascular and blood vessel invasion was observed within the tumor tissue.

After the surgery, a follow-up chest CT and abdomino-pelvic MRI were performed. The chest CT showed an increase in the size of the known lung lesions, growing to 8–17 mm. The abdominal MRI revealed that the previously identified lymph node near the lower third of the left kidney had enlarged to 18 mm.

Given the postpubertal-type teratoma with STM and pulmonary metastases, systemic therapy with capecitabine-oxaliplatin (CAPOX) plus bevacizumab was initiated in September 2023. Over 6 months (eight cycles) of treatment, the disease remained stable, leading to a transition to maintenance therapy with capecitabine-bevacizumab. The patient has since completed 24 cycles of this regimen. Staging assessments after 24 months continue to confirm stable disease.

### Patient 2

A 35-year-old male presented to our hospital with right subcostal pain radiating to the stomach and lumbar region in April 2007. Abdominal ultrasound and CT revealed a 110 × 70 mm mass located between the left kidney and the vertebral column. A chest CT showed no evidence of thoracic tumors. Core biopsy sampling yielded only necrotic tissue. Despite this, surgical removal was performed.

**FIGURE 2:**
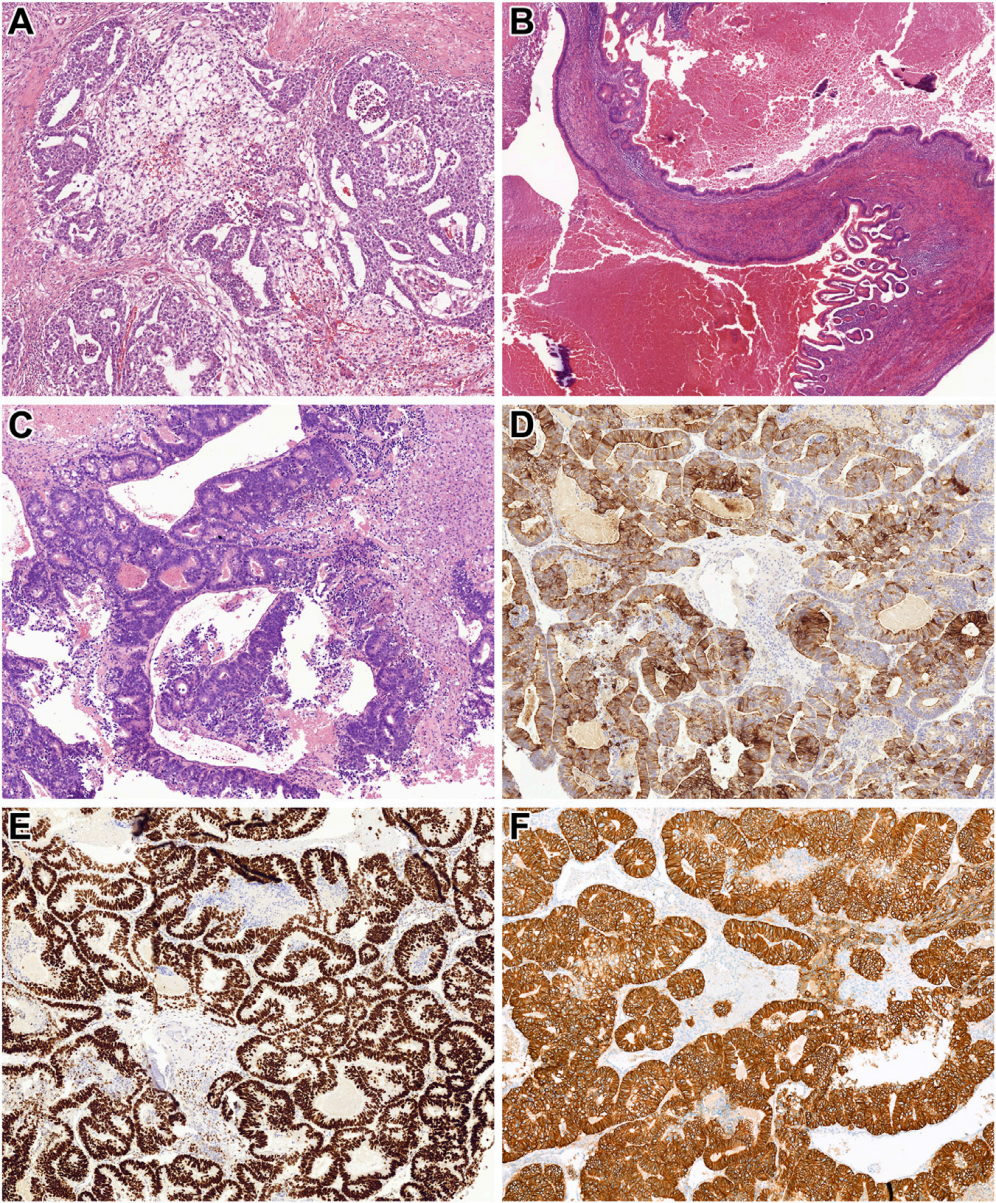
Histopathological features of *Patient 2’s* tumors. **(A)** Metastatic components identified in the retroperitoneal lymph nodes included embryonal carcinoma and microcystic yolk sac tumor (HE, x200). **(B)** The orchiectomy specimen revealed a mass of foreign tissue consistent with postpubertal-type teratoma. This image shows an area lined by intestinal-type epithelium (HE, x100). **(C)** The laminectomy specimen demonstrated an intestinal-type adenocarcinoma. Tumor cells formed atypical tubules with luminal necrotic debris (HE, x200). **(D,E)** Immunohistochemical staining showed diffuse positivity of the tumor cells for CK20 and SATB2, supporting intestinal differentiation (HE, x200 for both). **(F)** Additionally, strong and diffuse membranous expression of HER2 was observed in the tumor cells (HE, x200).

During the procedure, the tumor was found to be invading the abdominal aorta, necessitating partial resection. Histopathological analysis of the surgical specimen revealed a germ cell tumor comprising embryonal carcinoma and yolk sac tumor (Figure 2A). Based on these findings, a testicular ultrasound was conducted, identifying a 20 mm tumor in the left testis. Consequently, a left-sided orchiectomy was performed, and histological examination confirmed a postpubertal-type teratoma (Figure 2B). No other TGCT subtype was seen.

At the time of diagnosis, serum tumor markers were elevated, with an AFP level of 1,163.73 ng/mL and LDH at 3170 U/L. The patient underwent four cycles of BEP (bleomycin, etoposide, and cisplatin) chemotherapy starting in September 2007, which was well tolerated. Following treatment, AFP levels normalized (9.97 ng/mL), while LDH remained slightly elevated (620 U/L).

Post-treatment staging identified a residual retroperitoneal mass measuring 100 × 43 × 23 mm. A salvage retroperitoneal lymphadenectomy was performed, and histopathological examination revealed necrotic tissue without viable tumor cells. After surgery, all tumor markers returned to normal ranges (AFP: 8.39 ng/mL; LDH: 338 U/L). A postoperative abdominal CT identified a 35 × 20 mm lesion in the left renal hilum, which was determined to be a lymphocele. The patient was placed under oncological surveillance for 8 years, during which the lymphocele remained stable. Given the unusual nature of the case, follow-up was conducted according to a rigorous protocol. During the first year, chest X-ray, abdominal MRI, and testicular cancer–specific tumor marker assessments were performed every 8 weeks. In the second year, chest X-ray, abdominal MRI, and tumor markers were performed every 2 months; every 3 months in the third year; every 4 months in the fourth year; every 6 months in the fifth year; and annually thereafter until 2015, resulting in 8 years of total follow-up. Throughout this period, the lymphocele remained radiologically stable. Follow-up was eventually discontinued due to the patient being lost to follow-up.

Eight years later, in December 2023, the patient returned with complaints of lower back pain. A bone scintigraphy revealed a soft tissue mass with L2 vertebral destruction. An abdominal CT demonstrated a 70 × 55 × 47 mm tumor in the left renal hilum infiltrating the vertebral column. A biopsy of the mass identified an intestinal-type adenocarcinoma. Gastroscopy and colonoscopy were performed, but no tumors were found in the gastrointestinal tract. The patient’s CEA level was mildly elevated (13.6 ng/mL), and PET/CT detected no additional lesions.

Given these findings, the patient underwent debulking surgery and spinal stabilization. Histopathological analysis of the resected specimen confirmed an intestinal-type adenocarcinoma (Figures 2C–E), consistent with the initial biopsy results. FISH and NGS, performed as detailed in *Patient 1*, identified chromosome 12 overrepresentation leading to a final diagnosis of postpubertal-type teratoma with STM. Moreover, NGS identified pathogenic mutations in *TP53* and *PIK3CA* genes and amplification of *KRAS*, *CCND2*, *CDKN1B*, and *ERBB2*. The first three findings were in harmony with the FISH observations, while the last genetic change was supported by HER2 IHC (Figure 2F), too.

Despite receiving 10 × 3 Gy radiotherapy for the residual tumor, disease progression was observed in the retroperitoneum. The patient was subsequently treated with eight cycles of CAPOX combined with bevacizumab. As of the last follow-up, the disease has remained stable for 12 months.

## Discussion

STM rarely appears in TGCTs with an approximate incidence of 3%–6% in the primary and 8% in the metastatic tumors [3, 4]. Additionally, STM is found in about 20% of late recurrences and frequently after chemotherapy [5]. Histologically, the vast majority of STM cases is associated with postpubertal teratoma, and alternatively it may be linked to yolk sac tumor [6–8]. The exact pathogenesis of STM remains unclear and necessitates further research. Some authors suggested that chemotherapy is an important trigger [9].

By definition, a STM must occupy at least 0.5 cm of area in a histological section to qualify for diagnosis [9]. While carcinomas and sarcomas are the most common histological subtypes within STMs, other malignancies -including embryonic neuroectodermal tumors (ENET), nephroblastoma, and hematologic neoplasms- have also been reported [10, 11]. In a study of 30 testicular tumors with STM, Lobo et al. identified adenocarcinoma and ENET as the most frequent subtypes [4]. Similarly, Magers et al., analyzing 124 germ cell tumors with STM, found sarcomas to be the predominant histology [7].

In our experience, both patients presented with intestinal-type adenocarcinoma, NOS morphology, which is the most frequent histological subtype of the colorectal carcinoma. Notably, one case developed within the testis, while the other was identified in metastatic sites. Each case posed significant diagnostic challenges. In the first patient, STM was the only observed component, leading to an initial impression of a metastatic adenocarcinoma. Although testicular metastases are rare, adenocarcinomas -typically of gastrointestinal, pancreatic, or pulmonary origin- are the most commonly reported tumor types in such settings [12]. Nova-Camacho et al. analyzed 157 patients with testicular metastases and found that 46% had metastatic adenocarcinoma [13].

Further complicating the diagnostic process, the first patient was 63 years old at the time of diagnosis, an age at which TGCTs are uncommon [12, 14]. This unusual age profile, combined with the absence of typical germ cell morphology and the STM’s dominant appearance, highlighted the importance of thorough histopathological evaluation and the consideration of STM in the differential diagnosis of testicular and metastatic adenocarcinomas in older patients.

In the second patient, the STM developed at a metastatic site, specifically, in a retroperitoneal mass. Both patients exhibited postpubertal-type teratoma components in their primary tumors, and both initially presented with retroperitoneal metastases. According to the literature, the interval between the diagnosis of TGCT and the appearance of STM varies, with a reported median of approximately 70 months [15]. Notably, carcinomatous STMs tend to emerge later than sarcomatous ones [7]. In alignment with these observations, STM appeared 192 months after orchiectomy *in Patient 2*.

Given such extended latency periods and the unusual localization of the STM -especially when exhibiting adenocarcinoma morphology- other, more common primary tumors must be considered in the differential diagnosis. Consequently, a comprehensive diagnostic approach, incorporating the patient’s medical history, is critical. Immunohistochemistry alone cannot reliably distinguish between an STM with adenocarcinoma morphology and a primary adenocarcinoma unrelated to TGCT. In these cases, excluding alternative primary tumors is essential, and cytogenetic analysis of chromosome 12 provides a valuable diagnostic tool [16].

Since STM in TGCT arises from germ cell neoplasia *in situ*, it typically harbors chromosome 12 abnormalities, including gain and isochromosome 12p [17]. These alterations can be detected using molecular techniques. FISH is a relatively fast and reliable method for identifying such changes and is particularly useful in limited tissue samples. However, poor signal quality can complicate interpretation, posing challenges to the accuracy of the analysis.

NGS is also a suitable method for evaluating chromosome 12 alterations. Based on elevated copy number variations of genes located on chromosome 12, either full chromosomal gain or gain of the short arm can be inferred. However, NGS requires high-quality DNA for reliable analysis. Moreover, depending on the size of the gene panel used, NGS can identify additional pathogenic mutations, some of which may have therapeutic relevance. In *Patient 1*, who had a chemotherapy-naïve tumor, NGS revealed pathogenic mutations in *CTNNB1* and *STK11*. The *CTNNB1* gene encodes β-catenin, a key component of the Wnt signaling pathway [18]. While this pathway is activated in the vast majority of CRCs, the activation typically occurs via deleterious mutations in the *APC* gene (70%–80%), whereas activating mutations in *CTNNB1* are relatively rare (3%–10%) [19, 20]. The *STK11* gene is associated with Peutz-Jeghers syndrome when present as a germline mutation [21], which confers an increased risk of CRC; however, *STK11* mutations are exceptionally rare in sporadic CRC (<1%) [22]. Additionally, this tumor harbored high-level *MDM2* amplification, an alteration more commonly observed in soft tissue tumors [23]. Importantly, *MDM2* amplification represents a potential therapeutic target, with several clinical trials currently investigating MDM2 inhibitors [24].

*Patient 2’s* tumor harbored *TP53* mutation, a common alteration in CRC, although currently without direct therapeutic implications [19]. Furthermore, this tumor also carried a *PIK3CA* mutation and *ERBB2* (*HER2*) amplification. While *PIK3CA* is considered actionable primarily in hormone receptor-positive breast carcinomas [25], *ERBB2* amplification is targetable with HER2-directed therapies, offering a potential therapeutic avenue [26].

According to the literature, intratesticular STM does not significantly affect patient outcomes [27]. However, the presence of STM at metastatic sites is associated with a worse prognosis [28]. Lobo et al. reported that all patients in their cohort with STM confined to the primary tumor were alive, whereas 66.7% of those with STM arising in metastases or at relapse died from disease-related complications [4]. The histological subtype of STM does not appear to influence the prognosis [29]. The most favorable outcomes are achieved with complete surgical resection, although histological grade remains an important prognostic factor [30].

In cases where complete resection is not feasible, poor prognostic indicators include late relapse, advanced stage, incomplete resection, ENET histology, a higher number of chemotherapy cycles, and an extragonadal primary tumor [4].

In order to select the most appropriate treatment for a patient with a STM, it is essential -not only from a histopathological standpoint but also from a clinical perspective- to perform comprehensive differential diagnostic evaluations. Currently, there is no standardized diagnostic protocol for the work-up of these patients [31]. Based on the presenting clinical manifestations, it may be warranted to rule out primary tumors of the appendix, colon, stomach, pancreas, lung, or prostate from a differential diagnostic standpoint. In such cases, beyond the standard work-up typically performed for primary testicular tumors (including testicular ultrasound, chest-abdomen-pelvis CT, and testis-specific tumor markers), further diagnostic procedures are recommended -such as gastroscopy, colonoscopy, and, when indicated, endosonography or bronchoscopy- along with additional tumor marker tests (e.g., CEA, CA19-9, CA125, CA72-4, PSA). Accordingly, in all our cases presented here, the aforementioned differential diagnostic evaluations were conducted, and alternative primary tumor origins were excluded.

In cases of STM arising in the testis, selecting optimal treatment and ensuring long-term disease management pose significant challenges for clinicians. Reports in the literature regarding the treatment of similar patients remain limited, and there are no clear treatment guidelines available [11].

For our cases, treatment modalities were selected following multidisciplinary team discussions and an extensive review of the literature. It is well known that TGCTs are generally sensitive to platinum-based chemotherapy [11, 31]. However, this sensitivity often does not apply when STM transformation occurs [32]. Therefore, the choice of chemotherapy should be guided primarily by the histological subtype of somatic malignancy [11].

We conducted a comprehensive review of the literature focusing on testicular tumors associated with STM, with an emphasis on reported diagnostic approaches and therapeutic strategies. We identified 12 case reports (Table 2), 10 of which involved non-metastatic primary tumors [33–36, 39–43], while 2 were already metastatic at diagnosis [37, 38]. In four of the 12 cases, primary somatic-type malignant transformation was identified. All of these were locally or locoregionally advanced. Following initial orchiectomy, two of these patients underwent retroperitoneal lymph node dissection (RPLND), and one received adjuvant chemotherapy (VAC regimen) post-RPLND [34–36, 39].

**TABLE 2:** Reported postpubertal-type teratomas with somatic-type malignancy in the literature.

ID	Age at STM	Clinical stage primary TT	Histology of orchiectomy/primary RPLND	Adjuvant/definitive treatment of primary TT	Localization of STM	Size	Treatment	Late STM histology	Survival since STM (months)	Status
1.	50	IIA	PTT (50%), EC (30%), YST (15%)	1x EP – suspended due to poor tolerance	Retroperitoneum (6 years later)	13 × 13 mm	RPLND	ADC	NA	NA [33]
2.	21	IIA	Orchiectomy::PTT with STM of embryonal RMS and ChS RPLND: RMS, ChS, undifferentiated spindle cell sarcoma	RPLND	Primary testicular with metastasis to the retroperitoneum	20 mm	NA	NA	12	NED [34]
3.	38	IA	PTT with STM of RMS	Follow-up	Primary testicular	NA	NA	NA	NA	NA [35]
4.	43	IA	50% S, 50% PTT with STM of ChS	Follow-up	Primary testicular	NA	NA	NA	6	NED [36]
5.	46	IIIB	YST	4xBEP	Retroperitoneum (18 years later)	15 mm	RPLND	ADC	12	NED [37]
6.	40	IIIA	S	3xBEP	Retroperitoneum (9 years later)	50 × 60 mm	3xVIP followed by RPLND	MPNST	6	NED [38]
7.	40	IIA	Orchiectomy: PTT with STM of RMS RPLND: PTT	RPLND followed by 9xVAC	Primary TT	NA	NA	NA	NA	NA [39]
8.	51	IB	S	Follow-up	Inguinal region (1 year later) followed by multiple retroperitoneal metastasis	150 × 70 mm	RPLND, followed by 4x EP, followed by 4xBEP	Undifferentiated pleomorphic sarcoma	40	NED [40]
9.	47	IA/IIIA	Orchiectomy: EC (50%), PTT (45%), YST (5%) RPLND: PTT 18 months later: mediastinal lymph node resection: PTT	3xBEP followed by RPLND followed by mediastinal lymph node resection	Retroperitoneum (19 years later)	134 × 119 mm	RPLND followed by 1xBEP	ADC and low-grade LMS	24	AWD [41]
10.	39	IIA	Orchiectomy: EC RPLND: PTT	3xBEP followed by RPLND	Lung (13 years later)	76 × 38 mm	3x NS Cht followed by pneumonectomy	ADC	108	NED [42]
11.	58	IA	S	Follow-up	Pelvic region (10 years later)	100 mm	1x NS Cht followed by tumor resection	RMS, GBM, ADC	6	NED [42]
12.	59	IIB	Orchiectomy: S, EC, ChC RPLND: tumor free	RPLND followed by 8xBEP	Right gluteal region (28 years later)	120 × 90 × 80 mm	RT: 25 × 2Gy	Poorly differentiated carcinoma	12	AWD [43]

STM indicates somatic-type malignancy; TT, testicular tumor; RPLND, retroperitoneal lymph node dissection; ADC, adenocarcinoma; NA, not available; PTT, postpubertal-type teratoma; EC, embryonal carcinoma; YST, yolk sac tumor; EP, etoposide and cisplatin; RMS, rhabdomyosarcoma, ChS, chondrosarcoma; NED, no evidence of disease; S, seminoma; BEP, bleomycin, etoposide, and cisplatin; VIP, etoposide, ifosfamide, and cisplatin; MPNST, malignant peripheral nerve sheath tumor; VAC, vincristine, actinomycin-D, and cyclophosphamide; LMS, leiomyosarcoma; AWD alive with disease; NS Cht, not specified chemotherapy; GBM, glioblastoma; ChC, choriocarcinoma; RT, radiotherapy.

In the remaining eight cases, STM was identified as a late relapse. These recurrences occurred on average 13 years (range: 1–28 years) after the initial diagnosis. Of the eight late relapses, four presented in the retroperitoneal region [33, 37, 38, 41], while the others involved the inguinal [40], pulmonary [42], pelvic [42], or gluteal regions [43]. All four retroperitoneal relapses were treated with curative-intent RPLND; two of these patients also received testis-specific chemotherapy regimens (BEP and VIP). In the cases involving STM relapse in the inguinal, pulmonary, pelvic, and gluteal regions, treatment was guided by the extent of disease and consisted of curative/definitive therapy (radical surgery and/or radiotherapy); in two cases, unspecified chemotherapy was administered pre- and/or postoperatively (Table 2).

Both patients we present were diagnosed with metastatic disease, for which neither surgical nor other curative treatment options were available, and only palliative systemic oncologic therapy could be considered. Based on available data, we concluded that the clinical behavior of intestinal-type adenocarcinoma is most closely aligned with that of colorectal carcinoma, and possibly ovarian mucinous carcinoma. According to current NCCN and ESMO guidelines, platinum-based chemotherapy in combination with a VEGF inhibitor is a recognized therapeutic option for both tumor types [44–47]. Consequently, for our patients, we selected combination therapy with CAPOX plus bevacizumab. Following confirmation of stable disease at 9 months, treatment was de-escalated to maintenance therapy with capecitabine and bevacizumab.

Further therapy is always a matter of consideration, especially in such rare cases. In the event of disease progression, we suggest switching back from maintenance therapy by reintroducing the oxaliplatin agent into the regimen. In case of further progression or potential oxaliplatin intolerance, a thorough review of the current literature is recommended to explore additional therapeutic options, including targeted therapy in case of a targetable mutation, or switching to a chemotherapy combination containing irinotecan, as histology suggests that treatment can essentially follow the therapeutic algorithm for colorectal adenocarcinoma.

However, it must be noted that data on the efficacy of targeted therapy remain limited. Most of the published evidence is restricted to case reports, including one describing failed immune checkpoint inhibitor therapy [48]. The rarity of these tumors poses a significant challenge to the design and execution of prospective clinical trials. Nevertheless, comprehensive genomic profiling may reveal potential therapeutic targets, as demonstrated by Bremmer et al., who investigated the genomic features of 30 TGCTs with STM and identified 14 actionable genetic alterations [49].

Before the conclusions, we would like to briefly discuss the pathological entities to be considered in the differential diagnosis. As previously mentioned, STM can arise either intratesticularly or in metastatic sites, and in STM the most frequently observed components are various sarcomas and carcinomas [4, 5]. Nevertheless, melanoma [48], hematologic malignancies [28], and nephroblastoma-like tumors arising in the background of a teratoma have also been described [50]. When STM presents intratesticularly, the primary differential diagnostic consideration is metastasis. This can be excluded based on clinical information and the presence of GCNIS, although the latter is not always assessable.

If the morphology of STM corresponds to adenocarcinoma, among germ cell tumors yolk sac tumor and, less frequently, choriocarcinoma should be considered [8, 51]. Both of these tumors are hormone-producing: yolk sac tumors secrete AFP, while choriocarcinomas secrete beta-hCG [52]. In contrast, adenocarcinomas are typically non-secretory [52]. These entities can also be distinguished immunohistochemically, as yolk sac tumors are SALL4- and FOXA2-positive, while choriocarcinomas show beta-hCG expression [51]. Adenocarcinomas are generally negative for these markers, with the exception of hepatoid adenocarcinoma, which is FOXA2-positive [53]. In the context of intratesticular tumors, ovarian-type tumors should also be considered; however, these do not harbor chromosome 12 abnormalities [54].

When STM develops in a metastatic site, the correct diagnosis is strongly influenced by the location of the metastasis and the availability of clinical information [4]. We emphasize the importance of the latter, as in its absence the diagnostic process may become unnecessarily prolonged, and an excessive number of immunohistochemical and molecular pathological tests may be performed [55]. We also highlight that, in cases of metastatic TGCT associated with STM, the assessment of chromosome 12 abnormalities is essential for establishing the correct diagnosis.

We presented two cases of TGCT associated with STM, both demonstrating histological features of intestinal-type adenocarcinoma and harboring chromosome 12-related anomalies. In one case, STM was present at the time of diagnosis, while in the other, it developed during late recurrence. Complete surgical resection remains essential for achieving the best clinical outcome. STM is typically resistant to platinum-based chemotherapy; therefore, in metastatic settings, treatment should be guided by the specific histological subtype of the STM. Additionally, molecular genetic analysis is recommended to identify potential therapeutic targets and to support the histopathological diagnosis.

## Data Availability

The raw data supporting the conclusions of this article will be made available by the authors, without undue reservation.
